# The Unique Impact of COVID-19 on Human Gut Microbiome Research

**DOI:** 10.3389/fmed.2021.652464

**Published:** 2021-03-16

**Authors:** Ella Burchill, Eva Lymberopoulos, Elisa Menozzi, Sanjay Budhdeo, James R. McIlroy, Jane Macnaughtan, Nikhil Sharma

**Affiliations:** ^1^Faculty of Life Sciences and Medicine, King's College London, London, United Kingdom; ^2^Department of Clinical and Movement Neurosciences, Institute of Neurology, University College London, London, United Kingdom; ^3^Centre for Doctoral Training (CDT) AI-Enabled Healthcare Systems, Institute of Health Informatics, University College London, London, United Kingdom; ^4^National Hospital for Neurology and Neurosurgery, University College London Hospitals National Health Service (NHS) Foundation Trust, London, United Kingdom; ^5^EnteroBiotix, Aberdeen, United Kingdom; ^6^Institute for Liver and Digestive Health, University College London, London, United Kingdom

**Keywords:** COVID-19, gut microbiome, microbiome research, faecal microbiota transfer, clinical trials

## Abstract

The coronavirus (COVID-19) pandemic has disrupted clinical trials globally, with unique implications for research into the human gut microbiome. In this mini-review, we explore the direct and indirect influences of the pandemic on the gut microbiome and how these can affect research and clinical trials. We explore the direct bidirectional relationships between the COVID-19 virus and the gut and lung microbiomes. We then consider the significant indirect effects of the pandemic, such as repeated lockdowns, increased hand hygiene, and changes to mood and diet, that could all lead to longstanding changes to the gut microbiome at an individual and a population level. Together, these changes may affect long term microbiome research, both in observational as well as in population studies, requiring urgent attention. Finally, we explore the unique implications for clinical trials using faecal microbiota transplants (FMT), which are increasingly investigated as potential treatments for a range of diseases. The pandemic introduces new barriers to participation in trials, while the direct and indirect effects laid out above can present a confounding factor. This affects recruitment and sample size, as well as study design and statistical analyses. Therefore, the potential impact of the pandemic on gut microbiome research is significant and needs to be specifically addressed by the research community and funders.

## Introduction

Coronavirus disease 2019 (COVID-19) is caused by severe acute respiratory syndrome (SARS) coronavirus 2 (SARS-CoV-2) and has resulted in a global pandemic, as well as subsequent restrictions of public and private life. While clinical trials worldwide have been challenged as a consequence, there are unique implications for the rapidly evolving gut microbiome research.

The gut microbiome is the vast diverse population of an estimated 100 million−100 trillion microorganisms and their genes that populate the gastrointestinal tract (1). Through complex pathways, they play essential roles in the immune and metabolic pathways, thereby influencing maintenance of health and the pathogenesis of disease (2). This complex system can be disturbed by disease or lifestyle changes, mechanisms that become highly relevant in the context of the COVID-19 pandemic.

**Figure 1:**
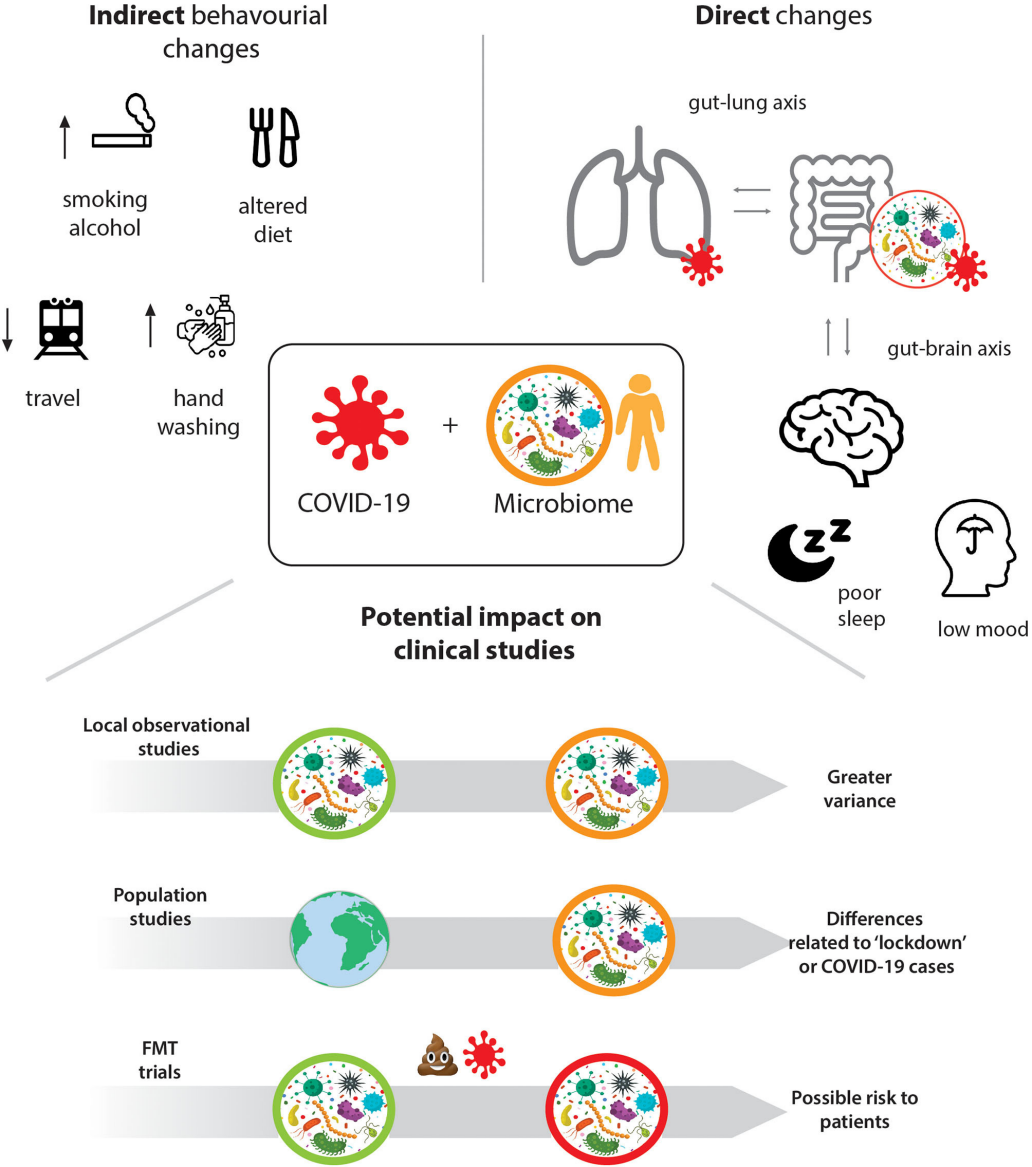
Direct and indirect impact of the COVID19 pandemic on human gut microbiome studies.

We propose that there are direct interactions between the gut microbiome and COVID-19, as well as indirect associations through the lifestyle changes induced by lockdowns and increased hygiene (see Figure 1) that need to be considered for ongoing and future microbiome studies. These range from experimental and observational, to longitudinal and population studies, as well as clinical trials using Faecal Microbiota Transplantation, FMT. We highlight how recruitment to studies, representativeness of samples, as well as the collection, storage, and analysis of stool samples are affected and how these effects can be mitigated through efficient study design, additional and rigorous statistical analysis, and collective effort.

## Direct Interactions Between SARS-CoV-2 and the Gut Microbiome

Increasingly, evidence shows an interaction between COVID-19 and gut microbiota homeostasis. Interactions between a healthy host and microbiota are extensive. They involve regulation of the innate and adaptive immune system (3), as well as maintenance of gut immune homeostasis and have disease-modifying potential (4). Additionally, the role of the gut microbiota is implicated in several lung diseases, with an indication of bidirectional communication termed the “gut-lung axis” (5). While this literature is rapidly growing, we provide a high-level overview.

The gut microbiome appears to contribute to the course and severity of COVID-19. A disrupted gut microbiome (gut dysbiosis) can be understood in terms of loss of beneficial microbes, proliferation of potentially harmful microbes, and reduction of microbial diversity (6). This leads to epithelium breakdown and inflammation, which have been shown to increase levels of angiotensin-converting enzyme 2 (ACE2), the target of SARS-CoV-2. Additionally, increased gut permeability can lead to pro-inflammatory bacterial products to leak out and circulate systemically, triggering inflammatory cascades (7). A specific gut microbiota composition may predispose healthy individuals to severe COVID-19 infections; increased levels of pro-inflammatory bacterial species correlated with elevated levels of pro-inflammatory cytokines and increased disease severity. Disruptions to the bidirectional microbiome-immune system dialogue are thought to be the cause of chronic inflammatory conditions, such as ulcerative colitis, and acute systemic multi-organ dysfunction, often accompanied by abnormal cytokine production. Therefore, a disrupted gut microbiome may also contribute to increased pro-inflammatory cytokine production (“cytokine storm”), known to worsen severity of SARS-CoV-2 infection (8). Proteomic and metabolomic profiling has shown progression to severe COVID-19 infection can be predicted both in infected patients and in healthy individuals (9). Furthermore, elderly and immunocompromised populations are known to have reduced microbiota diversity. Since many of these vulnerable patients have had worse clinical outcomes for COVID-19, this strengthens the possibility that the gut microbiome is affecting clinical progression. Reduced gut microbiome diversity may therefore be useful as a predictive biomarker of COVID-19 severity (10).

SARS-CoV-2 ribonucleic acid (RNA) has been found in COVID-19 patients' faeces (11, 12), implying transmission of SARS-CoV-2 could include faecal-oral (13). Furthermore, a meta-analysis found gastrointestinal symptoms occurred in 17.6% of infected patients, and were more common in severe cases (14). Mechanisms underpinning gastrointestinal symptoms remain unclear but are thought to involve ACE2 receptors, which are highly expressed on intestinal epithelial cells (15), in particular the brush border membrane of small intestinal enterocytes. ACE2 gene expression has been shown to increase with age, potentially accounting for differential susceptibility to more severe disease (16). Xiao et al. reported significant infiltration of plasma cells and lymphocytes with interstitial oedema in a histological analysis of one patient's intestinal tract (17). ACE2 expression has been shown to be downregulated in SARS patients, leading to reduced absorption of tryptophan and decreased release of antimicrobial peptides (18). This can lead to gut dysbiosis and sustain virus survival (19). ACE2 modification of the microbiota may therefore account for the observed gastrointestinal symptoms (20).

Importantly, studies have shown that SARS-CoV-2 RNA can be detected from stool samples for up to 14 days following clinical resolution and a negative respiratory tract sample (11, 17, 21). These results align with articles reporting viral shedding in stool samples collected from patients suffering from infections caused by other human coronaviruses, such as SARS-CoV-1 and MERS-CoV (22). Although there are limitations to studies reporting SARS-CoV-2 viral shedding, including lack of detail on robustness of analytical methods implemented, and lack of clearly reported study designs, the results have clear potential implications for infection prevention control, as well as for the FMT field (see below). However, to what extent the viral RNA correlates with intact viral particles is currently unclear.

Regarding lung microbiota, only one study to date has found changes in microbiota composition in SARS-CoV-2 patients, finding more pathogenic bacterial strains compared to healthy controls (9). The SARS-CoV-2 microbial composition was similar to microbiome changes observed with other respiratory viruses such as influenza. It is not currently known how ecologically stable the gut microbiome is during COVID-19 progression. Evidence suggests an association between illness severity and microbiota diversity in mechanically ventilated patients (23); this may apply to severe COVID-19 cases requiring ventilation. Further microbiome disease-related changes have been found when considering complications of COVID-19, such as acute respiratory distress syndrome (ARDS). High-throughput sequencing of gut and lung microbiota indicate altered bacterial composition in ARDS patients (compared to patients without ARDS), these bacterial composition changes may correlate with COVID-19 outcomes (24).

## Indirect Effects of Pandemic on an Individual or Populations' Microbiome

The COVID-19 pandemic led many countries to enforce lockdowns and other measures to reduce virus transmission. Although these vary in form, they share the promotion of better hand hygiene, reduction in social interaction, travel limitations, and a shift towards working from home. There are several indirect effects of the pandemic that have the potential to impact the gut microbiome at a large scale.

A key message from governments across the world is the importance of hand hygiene. Indeed, there is now large-scale use of disinfectants and sanitisers across society. The contribution of the environment to the microbiome is recognised and the use of sanitizers now and in the future may affect the microbiome of several ecological niches in humans, animals, and environments (25). While this may directly impact an individual's microbiome due to reduced exposure to environmental microbiota, its effect may also be seen at a population level. Health campaigns can result in long term behavioural changes (26), implying that the impact of regular hand sanitisation on the gut microbiome may endure long after the pandemic has resolved.

All aspects of travel have been severely restricted during the pandemic. Not only has national and international travel been reduced or even stopped, but there is a lack of household mixing. Overall, this will have lessened the exposure to a range of external environmental microbiota. Pre-pandemic, international travellers had a higher proportion of Escherichia species and increased antimicrobial resistance genes (27). While it is known that travel is a modifying factor for the adult microbiome (28), we do not know the effect of an absence of travel on the microbiome. These changes in travel habits may have impacted on the individual and population microbiome that could last into the future if international and national travel restrictions remain in place. The long-term increase in home working also needs to be carefully considered.

The impact of diet and lifestyle on the microbiome is unquestionable. The sudden lifestyle changes induced by the COVID-19 pandemic have been shown to alter eating habits, exercise and everyday behaviours (29). The increase in working from home, stockpiling groceries due to restrictions in shopping will have altered an individual's diet and therefore impacted the microbiome (30). Whether this results in greater or less diversity is unknown and may vary depending on the population itself. The psychological and emotional pandemic responses may have increased likelihood of dysfunctional or altered eating habits (31). Beyond modulation of host immunity, gut bacteria can also impact metabolic health, with specific gut bacteria changes and gut dysbiosis observed in metabolic disorders such as obesity and diabetes, known to be diet associated (32). Additionally, malnutrition is a massively concerning problem particularly for children in low and middle income countries, caused by pandemic-related financial straits, changes to food availability, and the interruption of school, healthcare, and social protection services (33, 34). Apart from the immediate increase of wasting syndrome, this will also have longer-term effects on physical and mental health through changes to the gut microbiome, setting off cascades of maladaptive metabolic responses and impaired immunity (35). Moreover, malnutrition has been suggested to negatively impact the course and outcome of a COVID-19 infection (36).

There are several non-dietary lifestyle changes that have occurred as a result of the pandemic. Exercise habits have changed both in the positive and negative manner. This is worthwhile considering since exercise can itself modulate the gut microbiome, orthogonally to changes induced by diet (37). The pandemic has increased alcohol consumption and smoking habits (38), in populations—both known to modulate the oral, lung, and gut microbiota (39, 40). A more unexpected result of the pandemic is the increase in pet ownership, which in itself can impact on the human gut microbiome (41).

The psychiatric and psychological burden of the pandemic is yet to be determined but early reports suggest a profound population level shift. The bidirectional microbiota-gut-brain axis has an active role in affecting mood and behaviour, research suggests population-level relationships between the microbiome and mental health (42). Social isolation, growing financial insecurity, and fear of the virus combined with unfamiliar social and lifestyle restrictions constitute major socioeconomic stressors that can potentially challenge individual and collective well-being and mental health, thus impacting the gut microbiome. The full psychiatric impact of the pandemic is not yet elucidated, but the implications are thought to be significant (31, 43). The pandemic has also been reported to alter sleeping patterns and quality (44), which in turn can negatively affect mood, stress, and anxiety. Additionally, the circadian rhythm is known to have a bidirectional relationship with the gut microbiome—disturbances in the gut microbiome can affect sleep regulation (41), and disturbances in circadian rhythms can alter the gut microbiome (45). This relationship has in fact been proposed as the mechanistic link between sleep disruption and metabolic syndrome, which can lead to diabetes, cardiovascular disease, and cancer (45). Therefore, the long-term consequences of COVID-19 on mental health should be considered in the light that this may implicate further microbiome interaction and additional negative health consequences for the host.

A recent review brings these changes together with the hygiene hypothesis (46), which refers to the current shift in the human microbiome composition towards lower diversity and loss of ancestral microbes that has been brought about by increased hygiene, antibiotics, and urban living (47). Taking these two processes together, the authors predict a substantial reduction of microbiome diversity which might not be able to be compensated for by the communal microbiome. We support this view and while the authors focus on opportunities for research into factors affecting the microbiome, we want to highlight the issues these changes present for ongoing and forthcoming microbiome research and clinical trials.

## The Effect of COVID-19 on Microbiome Studies

### Experimental, Correlational, and Longitudinal Microbiome Studies

Due to wide-reaching effects of COVID-19 and its unique interaction with the microbiome, it is important to consider how representative of the target population participants have become. Characteristics of patients enrolled before, during and after the pandemic may now be systematically different (48). These characteristics extend at least to the microbiota composition and diversity, but there may be more subtle changes. Whilst it appears an elegant concept, the reality of defining pre-, during-, and post- pandemic periods may be prohibitively complex due to global variability in the timing of the pandemic. Additionally, national as well as individual adherence to specific measures to combat the pandemic, as outlined above, differed substantially which introduces potentially significant between-subject variability, especially for studies recruiting globally. This could bias microbiome analysis and subsequent application of these results, particularly if studies are not designed to compare the pre-, intra-, and post-pandemic time points. There are further demographic and socioeconomic disparities to consider in light of the fact that COVID-19 is disproportionately affecting minority ethnicities and elderly populations, which already are lesser represented categories in any clinical trials (49). For longitudinal studies, for example, larger study populations may now be required due to the pandemic acting as a confounder, whereby more participants are lost to follow up due to COVID-19 infection.

Structural, clinical, physician, patient barriers to clinical trial participation during the pandemic have been already identified in the oncology field, accounting for most of patients' non-participation (50). Appraising these barriers from the perspective of microbiome trials, it is evident they can limit the resulting demographic of participants recruited. Structural barriers such as transportation, travel cost, availability of child and elderly care, changes in working patterns and employment opportunities have all been affected by the pandemic. Clinical barriers have increased during the pandemic due to narrower eligibility criteria and stricter safety concerns. There is also potential increased risk of selection bias and drop-out associated with personal aversion towards sample collection (51), due to individual awareness of the presence of viral RNA in faeces. Increased rate of comorbidities secondary to COVID-19 pandemic, together with the need to screen people for comorbidities in addition to asymptomatic infections, represents another issue.

Physician attitudes may also have changed as a result of the pandemic. Concerns about patient's safety may be heightened, meaning physicians might be hesitant to encourage enrolment in a new microbiome study. Moreover, time spent in clinical appointments for giving information about trials, discussing risks/benefits with the patient, and collecting informed consent is now severely affected due to restricted face-to-face interactions. Indeed, the generalised shift towards telemedicine for consultations may make recruitment to trials more difficult. It is also important to consider patients' and participants' attitudes may have changed, due to indirect effects of the lockdown, heightened concerns about sampling collection, and reluctance to attend clinical appointments and clinical trials in person.

Finally, sample collection and processing needs to be considered. The pandemic is unlikely to significantly interfere with most gut microbiome sample collection. The microbiome population can be investigated e.g., with 16S rRNA gene sequencing, quantitative PCR, or shotgun metagenomic sequencing. These investigative approaches analyse faecal samples; which rely on reproducible sample collection, storage and processing (52). However, there are technical issues, including safety concerns of shipping biological samples, according to the category of UN 3373 “Biological Substance, Category B.” During lockdown, it is likely that sample transport, delivery, and storage have been delayed. It is advised that transportation time should be shortened as much as possible, to avoid undesirable microbial growth and decline of sample quality (53). Furthermore, faecal sample collection methods have been shown to affect the microbial community profile (52). If these have changed during the lockdown or have been adversely lengthened, this may have detrimental impacts on subsequent analysis and comparisons.

### Interventional Faecal Microbiota Transfer Trials

There is growing scientific and clinical interest in the use of FMT to treat a diverse range of diseases in addition to *Clostridium difficle* infections; it is now trialled for inflammatory bowel disease, cancer and neurological disorders. FMT involves delivery or infusion of stool from a healthy donor to a patient with the disease of interest and presumed gut dysbiosis. In the UK, the MHRA has defined this as a pharmaceutical intervention and it is therefore subject to the same regulatory framework.

The presence of SARS-CoV-2 RNA in the stool of infected individuals raises the suggestion of virus transmission via FMT, the risks of which are currently unknown (13). It is also unclear if asymptomatic but serologically positive individuals can transmit the virus if viral particles are detected in faeces (54). The Food and Drug Administration (FDA) has subsequently advised additional safety protections for FMT are necessary, recommending stool used should have been originally donated before 1st December 2019. This clearly limits shelf-life of samples. Donor stool may have altered before, during and after COVID-19, which impacts trials ongoing or due to begin recruiting imminently. This suggests all stool samples should now be routinely tested and stringently screened for COVID-19 (55), which may lead to stricter exclusion criteria for stool donors. As the COVID-19 status of the donor may affect its recipient adversely, is it more acceptable to adopt a single donor approach instead of several pooled donors, who may collectively carry higher virus risk. The COVID-19 status of the recipient is also worth considering, as recipients may be rendered more susceptible if their donor is COVID-19 negative. Is it more advantageous for a donor to have had the virus previously and potentially confer immune protection via IMT, or does this conversely put the recipient more at risk of developing COVID-19? Unanswered questions remain.

In disease-focused microbiome trials, the impact of COVID-19 on the disease should be considered upon recruitment of participants receiving FMT. The disease population may now be atypical, due to COVID-19 disease interactions and interruptions of regular treatment, and likely constitute a vulnerable population. Indeed, clinical trials often focus on vulnerable populations, who are more at risk from COVID-19. Specifically, there is growing interest in bidirectional interactions of the gut microbiota and neurodegenerative diseases, which constitute a diverse population, likely to be more vulnerable. For example, patients with Multiple Sclerosis, a common patient group for FMT trials (56) and a high-risk group for COVID-19 (due to wide-spread use of immunosuppressant medication), may have shielded extensively or had reduced face-to-face check-ups due to reduction of clinical services. This altered environment may have subsequently changed their microbiome composition, raising the question of how representative the sample now is of the wider MS patient population independent of the pandemic. The same applies to Motor Neurone Disease or Parkinson's Disease patients, whom are also increasingly the focus of FMT trials. Other risk factors for severe COVID-19 infection include hypertension, diabetes, and obesity. All are associated with changes to microbiota composition and diversity, posing the question of whether COVID-19 risk factors should be considered upon recruitment for microbiome trials.

Finally, FMT trials often utilise hazard ratios and primary endpoints, which may no longer be plausible if defined before the pandemic. Trials utilising imaging are likely to be delayed and restricted due to the pandemic. All are crucial considerations for future study analysis and interpretation. Possible mitigations include sophisticated covariate adjustments (57) for variable COVID-19 prognosis and trajectory. Double/debiased machine learning approaches may be indicated to distinguish primary outcomes and to perform formal statistical inference (58).

## Conclusion

In summary, the COVID-19 pandemic may impact several aspects of microbiome studies that need to be explored further. The direct interaction between the gut microbiome and the severity of COVID-19 infection is a highly active research area and we look forward to the future publications in the area. Additionally, we have explored the indirect effects on individual and population microbiome composition. To reduce the impact of these changes on microbiome studies, pre-, intra-, and post-pandemic microbiome reference libraries may be necessary to exclude potential COVID-19-related confounders and to assess for stability across these fluctuating time points. Funders in this area may consider specific calls in this area and a UK or international gut microbiome consortium may be needed to coordinate efforts.

The impact for trials is an immediate concern. For trials already underway, this—in addition to the baseline shift of microbiome abundance—may mean the trial is no longer sufficiently powered. An open data policy is recommended to mitigate this, although funders should be open to additional studies being required. Finally, FMT studies must consider potential COVID-19 transmission, and may need to account for the pandemic-related microbiome compositional changes in the analysis. To avoid pandemic-related confounds when assessing microbiota interactions with non-COVID-19 diseases or interventions, large study populations will likely be most useful. Additional testing of stool donors (e.g., for COVID-19 infection or antibodies), potential confounds (e.g., shielding), and open microbiome data will undoubtedly be required. Again, additional funding may be required to specifically address these points.

In summary, “COVID-19 is with us for the long haul, a marathon that we will run for months or years to come” (59). Current studies and future work needs to specifically address and account for these potential sources of change. There are other emerging areas that need to be considered such as the effect of “Long COVID” and multiple COVID-19 vaccinations which may also impact the gut microbiome studies. We must maximise utility of data already collected and reconsider how future trials can be protected. Lessons can be learnt from rapid progress achieved by clinical trials designed to research COVID-19, exposing certain aspects of trials that can be improved universally to benefit patients, researchers and clinicians. The microbiome community must work with funders to perform the necessary research to establish the actual impact of the pandemic.

## Author Contributions

EB and NS devised the idea, conceived the figure and wrote and edited the manuscript. EL, EM, SB, JMc, and JMa contributed to the manuscript. All authors contributed to the article and approved the submitted version.

## Conflict of Interest

JMc holds shares in and is an employee of EnteroBiotix Limited. He is a named inventor on patents relating to the gut microbiome. JMa holds shares in Yaqrit Ltd. The remaining authors declare that the research was conducted in the absence of any commercial or financial relationships that could be construed as a potential conflict of interest.
